# Disparities in Patterns of Preterm and Early Term Second Births Among Non‐Hispanic Black and White Mothers

**DOI:** 10.1111/ppe.70083

**Published:** 2025-11-16

**Authors:** Puneet Kaur Chehal, Maria Dieci, E. Kathleen Adams, Michael R. Kramer, Anne L. Dunlop

**Affiliations:** ^1^ Department of Family and Preventive Medicine, School of Medicine Emory University Atlanta Georgia USA; ^2^ Department of Health Policy and Management, Rollins School of Public Health Emory University Atlanta Georgia USA; ^3^ Center for Rural Health and Health Disparities, School of Medicine Mercer University Atlanta Georgia USA; ^4^ Department of Gynecology and Obstetrics Emory University School of Medicine Atlanta Georgia USA

**Keywords:** early term birth, preterm birth, previous birth, race disparities, second births, shortened gestational age

## Abstract

**Background:**

Early term births (37–38 weeks), like preterm births (< 37 weeks) are associated with increased infant morbidity, mortality, and risk of future preterm births. While racial disparities in preterm births are well documented, longitudinal patterns of early term and preterm births by maternal race remain underexplored.

**Objectives:**

To estimate the likelihood of second births that are preterm or early term, conditional on the gestational age category of the mother's first birth and maternal race.

**Methods:**

This population‐based cohort study used linked birth and hospital discharge records for non‐Hispanic (NH) Black and White mothers in Georgia with a first and second singleton live birth between 2011 and 2020. We examined the unadjusted distributions of second birth gestational age (< 32, 32–36, 37–38, ≥ 39 weeks) stratified by first birth gestational age category and maternal race. Adjusted relative risk ratios (RRRs) were estimated using multinomial logit models.

**Results:**

NH Black mothers delivered 31,768 births; NH White mothers delivered 58,113. Among mothers with a first preterm birth < 32 weeks, NH Black mothers had a higher likelihood of second births at < 32 (RRR 19.08, 95% CI 14.48, 24.98) than NH White mothers (10.17, 95% CI 7.00, 14.78) and had similar disparities for second births at 32–36 weeks. After early term first births, NH Black mothers had elevated risks of < 32 or 32–36 week births (RRRs 3.53, 95% CI 2.90, 4.30 and 2.88, 95% CI 2.64, 3.13 respectively) versus NH White mothers (1.73, 95% CI 1.41, 2.11 and 2.07, 95% CI 1.92, 2.22). Racial disparities extended to second births following full‐term first births and persisted after restricting the sample to non‐indicated first births.

**Conclusions:**

NH Black mothers face relatively elevated risks of shortened gestation in subsequent births, regardless of the gestational age of their first birth, including after early term or full‐term births.

## 1 Background

Preterm birth (< 37 weeks' gestation) remains a leading contributor to neonatal mortality in the U.S. [1] Increasing evidence also implicates early term births (37–38 weeks' gestation) in higher rates of infant mortality and morbidity [2, 3, 4]. Whereas preterm births accounted for fewer than 10% of US births in 2022, early term births accounted for nearly 30% [5].

Population‐based research demonstrates that preterm and early term births have overlapping maternal, fetal, and placental risk factors and similar placental pathologic features [6, 7, 8, 9]. In recognition of shared risk associated with shortened gestation, the American College of Obstetricians and Gynaecologists and the American Academy of Paediatrics advocate for prevention strategies that target both groups [10, 11, 12]. Like preterm birth, early term birth is associated with prior obstetric history and may signal elevated risk of shortened gestation in future pregnancies [9, 13, 14, 15, 16, 17, 18]. However, there is sparse research on the longitudinal patterns of early‐term and preterm births by maternal race [19, 20].

Persistent Black‐White disparities in birth outcomes reflect the complex social effects of race in the US [21]. Black mothers have higher rates of clinically indicated and spontaneous preterm births [22]. Longitudinal analysis offers a valuable lens for investigating these disparities by examining how prior birth outcomes influence future risk. Comparing gestational age outcomes across multiple births within the same mothers over time can yield insights into the maternal factors driving racial disparities in shortened gestational age [21]. In this study, we examined the risk of preterm and early term *second* births conditional on the gestational age category of *first* live births among non‐Hispanic (NH) Black and NH White mothers in Georgia. Georgia is a populous, racially diverse Southern state with consistently higher rates of preterm birth among NH Black mothers compared to NH White mothers (15.0% vs. 10.3% from 2021 to 2023) [23, 24].

## 2 Methods

### 2.1 Cohort Selection

In this retrospective cohort study, we use linked birth and hospital discharge record data from the Georgia Department of Public Health to identify live singleton births to Georgia‐resident mothers with two observable, sequential singleton live births—specifically, their first and second births—between 2011 and 2020 [18]. Fetal deaths were excluded due to their rarity (7.0 per 1000 live births) and distinct etiological profiles, consistent with prior related work [19, 20, 24]. Detail on the data and sample selection is illustrated in a flow diagram in Appendix S1.

We restricted the analytic sample to birth records with matched hospital discharge records. Birth records of non‐resident mothers, missing gestational age and potentially erroneously recorded, closely spaced births (< 180 days apart) were excluded. Variables in the birth record data indicating live versus stillbirth status and birth order were used to identify mothers' first and second live births [18].

We focused on live births to non‐Hispanic (NH) Black and NH White individuals, excluding those with missing or inconsistent race data across both births or for whom ethnicity was Hispanic or missing. If maternal race was missing for one birth but reported in the other, we imputed the missing value for race (*n* = 792). Ultimately, the final analytic sample included 179,762 births to 89,881 mothers (31,768 NH Black and 58,113 NH White) whose first two live sequential hospital births were observable in the data.

For subgroup analysis, we excluded mothers with *first* births that were clinically indicated deliveries to focus on early births that were likely spontaneous. Clinically indicated early births often arise from maternal or fetal complications—such as hypertensive disorders of pregnancy or placental insufficiency—and may signal distinct recurrence patterns relative to spontaneous early births [16, 25]. Following the literature, we used information from both birth certificates and hospital discharge records to classify early births as clinically indicated or not. Clinically indicated births were those with no documentation of premature rupture of membranes (PROM) or premature labor or tocolysis, but with documentation of artificial rupture of membranes, medical or surgical induction, or a caesarean delivery without onset of labor (Appendix S2) [13]. Excluding mothers with clinically indicated *first* births for subgroup analysis helps to better capture biologically driven recurrence risk among mothers with prior spontaneous early deliveries which make up the larger proportion of early deliveries, and enables more focused examination of race‐based disparities in spontaneous recurrence [21].

### 2.2 Exposure

The primary exposures were eight mutually exclusive categories based on maternal race (NH Black or NH White) and gestational age at *first* birth, categorised as < 32, 32–36, 37–38, or ≥ 39 weeks.

### 2.3 Outcome

The primary outcome was gestational age of *second* birth, categorised as < 32, 32–36, 37–38, or ≥ 39 weeks [18].

### 2.4 Covariates

Maternal sociodemographic covariates were taken from the second birth record unless otherwise noted. These included age group at first birth (< 18, 18–34, 35 ≥ (reference)), country of birth (not US or US (reference)), marital status (married, not married (reference)), *same* paternity information (yes, no (reference)) reported by the mother for the *first* and *second* births for father's year of birth, race, or ethnicity, educational attainment at *second* birth (no high school, high school only, some college or more (reference)), and Medicaid coverage (yes, no (reference)). We used hospital discharge records to construct an indicator for Medicaid insurance coverage at the second birth reflecting the state's Medicaid programs (traditional Medicaid, the Children's Health Insurance Program, and managed care programs). Medicaid is the publicly funded health insurance program in the US which has historically focused on low‐income pregnant women.

Maternal clinical risk factors were derived from International Classification of Diseases (ICD) 9 and 10 diagnostic or procedural codes in hospital discharge record data (Appendix S3) and included indicators for: smoking, prior caesarean delivery, any hypertension, urinary tract infection (UTI) without indwelling catheter, any diabetes, substance use, anaemia, pregnancy complicating mental disorder, and infection(s) (syphilis, gonorrhoea, other venereal diseases, other infectious and parasitic disease, tuberculosis, malaria, rubella, other viral disease, unspecified infection or infestation) [26, 27]. Both information from birth records and hospital discharge records was used to identify maternal smoking. Details on how variables were constructed are included in Appendix S2. Interpregnancy interval was either included on the birth record or calculated as the difference between the date of birth for the *second* birth, less gestational age for the *second* birth, and the date of birth for the *first* birth.

All models included fixed effects for the calendar year of the second birth to control for secular trends, such as clinical practice changes, coding shifts, or policy interventions such as the Affordable Care Act (ACA) or the 2011 introduction of Georgia's Medicaid waiver program Family Planning for Healthy Babies. Because of the small sample size of second births in 2011, our first year of data, we combined data from 2011 and 2012 into a single category for fixed effect estimation purposes.

### 2.5 Statistical Analysis

We first described unadjusted race‐specific distributions of *second* birth gestational age categories, stratified by the gestational age category of mothers' *first* birth. We then estimated adjusted multinomial logit models to jointly evaluate the likelihood for *second* birth gestational age categories conditional on all possible combinations of previous gestational age categories and the mother's race. The multinomial logit model compares the likelihood of each outcome to a designated referent outcome, which we set to *second* birth at full term. Thus, our estimates compare the likelihood of each of the comparative *second* birth gestational age outcomes (< 32, 32–36, or 37–38 weeks) relative to the likelihood of full‐term births (≥ 39 weeks) (the referent outcome), and how these relative likelihoods differ across groups categorised by *first* birth gestational age category and maternal race. The benefit of this approach includes increased statistical efficiency, prevention of the multiple comparisons problem, and ensures that the sum of probabilities for *second* birth gestational outcomes is one [28, 29].

As is necessary for categorical right‐hand‐side variables, we excluded one category (or, in this case, a combination) to be the reference, which we selected to be NH White mothers with a *first* full‐term birth. Thus, we compare all other combinations of race and *first* birth gestational category to mothers with NH White mothers with a *first* full‐term. The benefit of this approach is that we can compare estimates for NH Black and White mothers as all estimates are being compared to the same benchmark simultaneously: NH White women with a *first* full‐term birth.

We used model estimates to report adjusted relative risk ratios (RRRs). The RRRs quantify how being a part of a specific maternal race and *first* birth gestational category group (as compared to the reference group) affects the likelihood of *second* birth gestational category relative to the referent outcome, which is set to a *second* full‐term birth (≥ 39). An RRR greater than 1 means that being a part of the specific race and *first* birth gestational age group increases the likelihood of the respective comparative *second* birth gestational age category (either < 32, 32–36 or 37 weeks) relative to the referent outcome (≥ 39 weeks), i.e., making the comparison outcome more likely. Conversely, an RRR less than 1 indicates that being a part of the specific race and *first* birth gestational age group, decreases the likelihood of the comparison outcome relative to the referent outcome [28]. 95% confidence intervals were reported with model results and imputed sociodemographic statistics. All analyses were completed using Stata 18.5.

Models were adjusted to account for maternal sociodemographic indicators and maternal risk factors at *second* birth that are linked to shortened gestational age at delivery in the literature [13]. These factors are likely observable to providers, and holding them constant allows us to evaluate the *unique* and otherwise unexplained signal for future shortened gestational age at delivery associated with previous preterm or early term births, and whether there are race differences in this association that are not explained by differences in observable maternal characteristics.

### 2.6 Missing Data

As mentioned earlier (and shown in Appendix S1), mothers with missing or inconsistent race information, missing ethnicity information and in a few cases missing gestational age at delivery were excluded from the analytic sample. Missing sociodemographic characteristics were imputed using chained multiple imputation. There were no missing values for maternal risk factors as they were derived from hospital discharge records and only women with matched hospital discharge records are included in the sample. Levels of missingness for covariates and imputation output are reported in Appendix S4.

### 2.7 Ethics Approval

This study's protocol was reviewed and approved by the Emory University Institutional Review Board, IRB00112252. The IRB waived informed consent in accordance with the US Common Rule [30].

## 3 Results

**FIGURE 1:**
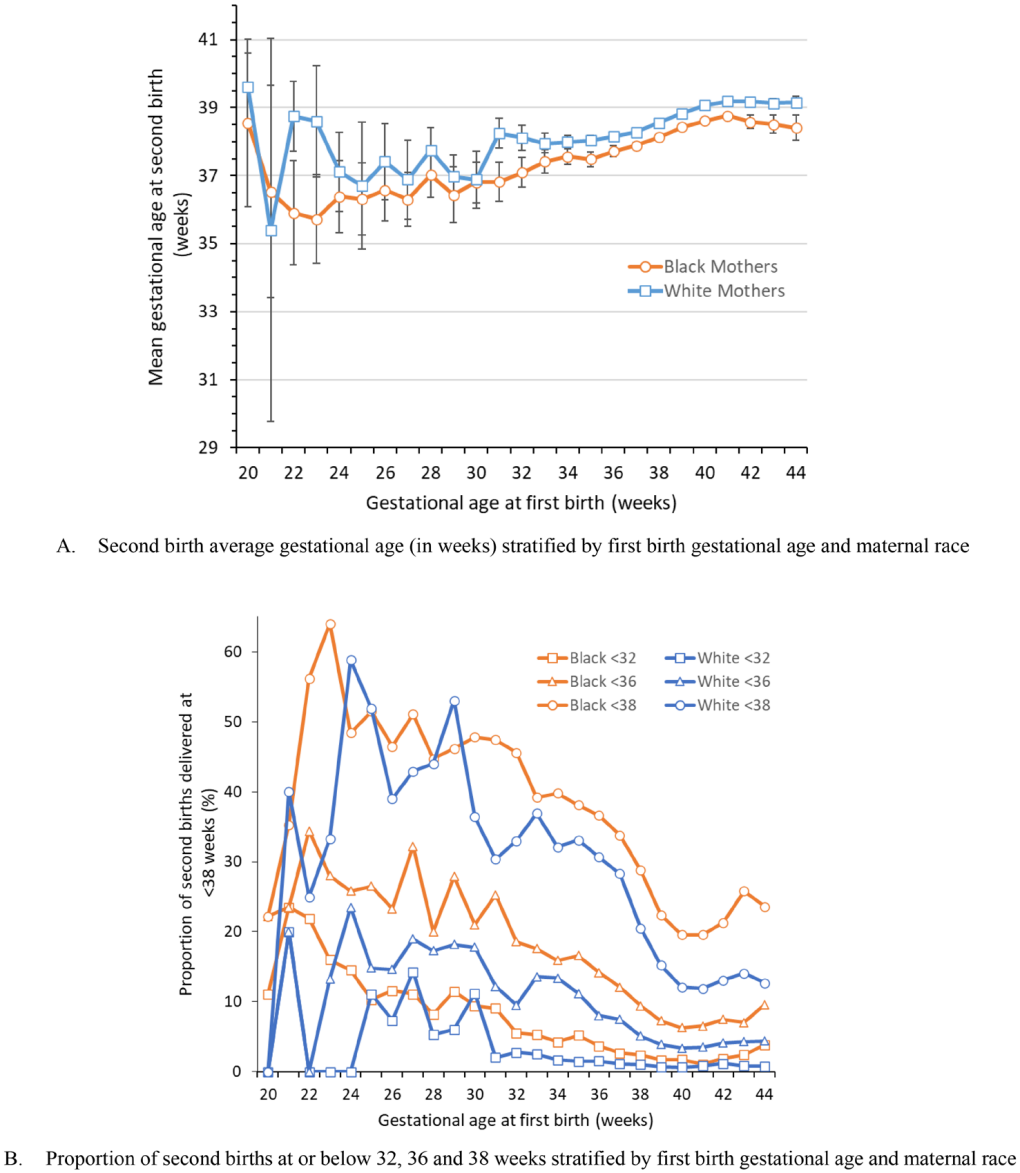
Distribution of gestational age of second births stratified by gestational age of first births and maternal race: Georgia, 2011 to 2020. (A) Second birth average gestational age (in weeks) stratified by first birth gestational age and maternal race. (B) Proportion of second births at or below 32, 36 and 38 weeks stratified by first birth gestational age and maternal race. The analytical sample is limited to births from mothers whose first two consecutive births were live and observable in Georgia between 2011 and 2020. The vertical lines denote the 95% confidence intervals for the mean gestational age of the second birth. Adapted from analysis originally published in Ananth, Misra, Demissie and Smulian [31].

Regardless of gestational age at *first* delivery, second births to NH Black mothers in Georgia are generally born earlier than *second* births to NH White mothers with the same gestational age at *first* delivery (Figure 1). Preterm *second* births were less common overall. Racial disparities in second early births were especially marked among mothers whose first births were early term. Among those with a first early term birth, NH Black mothers experienced higher unadjusted proportions of a subsequent preterm birth at either < 32 weeks and 32–36 weeks than NH White mothers (respectively 2.5% and 14.4% for NH Black; 1.1% and 9.9% for NH White) (Table 1) and were also less likely to have a full term *second* birth than White mothers (respectively 49.4% and 55.6%), although proportions of early term recurrence were similar across NH Black and White mothers (respectively 33.7% and 33.4%).

**TABLE 1:** Unadjusted probabilities of second birth gestational age categories stratified by first birth gestational age category and maternal race.

Gestational week at delivery of 1st birth	Gestational week at delivery of the 2nd birth																			
	Black mothers										White mothers									
	< 32		32–36		37–38		39+		Row total		< 32		32–36		37–38		39+		Row total	
	*n*	%	*n*	%	*n*	%	*n*	%	*n*	%	*n*	%	*n*	%	*n*	%	*n*	%	*n*	%
< 32	102	(11.4%)	200	(22.3%)	269	(30.1%)	324	(36.2%)	895	(100%)	36	(6.5%)	97	(17.4%)	169	(30.4%)	254	(45.7%)	556	(100%)
32–36	147	(4.4%)	652	(19.7%)	1104	(33.3%)	1413	(42.6%)	3316	(100%)	67	(1.7%)	645	(16.3%)	1347	(34.0%)	1901	(48.0%)	3960	(100%)
37–38	213	(2.5%)	1233	(14.4%)	2891	(33.7%)	4234	(49.4%)	8571	(100%)	140	(1.1%)	1277	(9.9%)	4315	(33.4%)	7179	(55.6%)	12,911	(100%)
39 ≥	319	(1.7%)	1840	(9.7%)	5201	(27.4%)	11,626	(61.2%)	18,986	(100%)	300	(0.7%)	2306	(5.7%)	9083	(22.3%)	28,997	(71.3%)	40,686	(100%)
Total	781	(2.5%)	3925	(12.4%)	9465	(29.8%)	17,597	(55.4%)	31,768	(100%)	543	(0.9%)	4325	(7.4%)	14,914	(25.7%)	38,331	(66.0%)	58,113	(100%)

*Note:* The analytical sample is limited to births from mothers whose first two consecutive births were live and observable in Georgia data between 2011 and 2020. The percentages reflect the proportion of mothers who give birth at each gestational age category in their second birth conditional on first pregnancy gestational age, or risk. Within each panel, the rows sum to 100%.

NH Black mothers also had higher rates of recurrent preterm births. Specifically, the proportion of a recurrent preterm birth at < 32 weeks was especially large for NH Black mothers compared to White mothers (11.4% vs. 6.5%). The proportion of *second* preterm births at 32–36 weeks after a *first* birth at < 32 weeks was also higher for NH Black mothers relative to White mothers, although the difference was smaller (22.3% vs. 17.4%). These disparities persisted among mothers with a *first* preterm birth at 32–36 weeks. Proportions of *second births* that were early term after a preterm birth (< 32 or 32–36 weeks) were more similar among NH Black (range across types of preterm births: 30.1%–33.3%) and NH White mothers (range across types of preterm births: 30.4%–34.0%).

Tables 2 and 3 show racial differences in the sociodemographic characteristics and maternal risk factors at *second* birth for mothers in our study sample. NH Black mothers were more likely to be younger at first birth, have lower educational attainment, be of immigrant status, and rely upon means‐tested health insurance coverage from Medicaid. While there were Black‐White differences in maternal clinical risk factors such as previous C‐section, smoking, drugs/alcohol abuse, iron deficiency, diabetes and hypertension, the differences were largely consistent across mothers with and without clinically indicated *first* births. There were, however, higher rates of *second* preterm or early term births among non‐indicated *first* births (bottom of Table 3) for mothers of both races.

**TABLE 2:** Maternal sociodemographic descriptive statistics stratified by maternal race.

Maternal characteristics	All mothers		White mothers		Black mothers	
Number of mothers	89,881	(100%)	58,113	(100%)	31,768	(100%)
Age at first birth						
Less than 18 years of age (%)	5417	(6.0%)	2025	(3.5%)	3392	(10.7%)
	(5277–5557)	(5.9%–6.2%)	(1939–2112)	(3.3%–3.6%)	(3284–3500)	(10.3%–11.0%)
Between 18 and 34 years of age (%)	80,785	(89.9%)	53,333	(91.8%)	27,452	(86.4%)
	(80,608–80,962)	(89.7%–90.1%)	(53,203–53,463)	(91.6%–92.0%)	(27,332–27,572)	(86.0%–86.8%)
35 years and older (%)	3679	(4.1%)	2755	(4.7%)	924	(2.9%)
	(3563–3795)	(4.0%–4.2%)	(2655–2855)	(4.6%–4.9%)	(865–983)	(2.7%–3.1%)
Education						
Has less than high school diploma (%)	7910	(8.8%)	4089	(7.0%)	3821	(12.0%)
	(7742–8077)	(8.6%–9.0%)	(3967–4210)	(6.8%–7.2%)	(3707–3936)	(11.7%–12.4%)
Has high school diploma (%)	25,538	(28.4%)	11,787	(20.3%)	13,751	(43.3%)
	(25,272–25,804)	(28.1%–28.7%)	(11,596–11,977)	(20.0%–20.6%)	(13,577–13,925)	(42.7%–43.8%)
Has some college education or more (%)	56,433	(62.8%)	42,238	(72.7%)	14,196	(44.7%)
	(56,149–56,718)	(62.5%–63.1%)	(42,027–42,449)	(72.3%–73.0%)	(14,022–14,370)	(44.1%–45.2%)
Birth country						
Born in the USA (%)	85,828	(95.5%)	56,216	(96.7%)	29,612	(93.2%)
	(85,706–85,950)	(95.4%–95.6%)	(56,132–56,301)	(96.6%–96.9%)	(29,524–29,700)	(92.9%–93.5%)
Born outside the USA (%)	4053	(4.5%)	1897	(3.3%)	2156	(6.8%)
	(3931–4175)	(4.4%–4.7%)	(1812–1981)	(3.1%–3.4%)	(2068–2244)	(6.5%–7.1%)
Marital status^a^						
Yes (%)	50,616	(56.3%)	44,057	(75.8%)	6559	(20.6%)
	(50,324–50,907)	(56.0%–56.6%)	(43,854–44,259)	(75.5%–76.2%)	(6418–6701)	(20.2%–21.1%)
No (%)	39,265	(43.7%)	14,057	(24.2%)	25,209	(79.4%)
	(38,974–39,557)	(43.4%–44.0%)	(13,854–14,259)	(23.8%–24.5%)	(25,067–25,350)	(78.9%–79.8%)
Medicaid^a^						
Yes (%)	43,577	(48.5%)	19,350	(33.3%)	24,227	(76.3%)
	(43,283–43,871)	(48.2%–48.8%)	(19,127–19,573)	(32.9%–33.7%)	(24,078–24,376)	(75.8%–76.7%)
No (%)	46,304	(51.5%)	38,763	(66.7%)	7541	(23.7%)
	(46,010–46,598)	(51.2%–51.8%)	(38,540–38,986)	(66.3%–67.1%)	(7392–7690)	(23.3%–24.2%)
Same reported father race^b^						
Yes (%)	87,417	(97.3%)	56,797	(97.7%)	30,620	(96.4%)
	(87,271–87,563)	(97.1%–97.4%)	(56,708–56,885)	(97.6%–97.9%)	(30,527–30,714)	(96.1%–96.7%)
No (%)	2464	(2.7%)	1316	(2.3%)	1148	(3.6%)
	(2318–2610)	(2.6%–2.9%)	(1228–1405)	(2.1%–2.4%)	(1055–1241)	(3.3%–3.9%)
Same reported father hispanic^b^						
Yes (%)	88,526	(98.5%)	57,200	(98.4%)	31,326	(98.6%)
	(88,449–88,603)	(98.4%–98.6%)	(57,140–57,261)	(98.3%–98.5%)	(31,278–31,373)	(98.5%–98.8%)
No (%)	1355	(1.5%)	913	(1.6%)	442	(1.4%)
	(1278–1432)	(1.4%–1.6%)	(853–973)	(1.5%–1.7%)	(395–490)	(1.2%–1.5%)
Same father birth year^b^						
Yes (%)	52,620	(58.5%)	36,179	(62.3%)	16,440	(51.8%)
	(52,241–52,998)	(58.1%–59.0%)	(35,929–36,430)	(61.8%–62.7%)	(16,197–16,683)	(51.0%–52.5%)
No (%)	37,262	(41.5%)	21,934	(37.7%)	15,328	(48.2%)
	(36,883–37,640)	(41.0%–41.9%)	(21,683–22,184)	(37.3%–38.2%)	(15,085–15,571)	(47.5%–49.0%)
Birth interval months^c^	33.96		32.88		35.95	
	(33.84–34.09)		(32.75–33.02)		(35.71–36.18)	

*Note:* The analytical sample is limited to births from mothers whose first two consecutive births were live and observable in Georgia between 2011 and 2020. Estimated or actual frequencies and proportions (in parentheses) of NH White and NH Black mothers' second births. 95% Confidence Intervals in brackets.

^a^ At second birth.

^b^ Comparing mother‐reported paternal characteristics at first and second births.

^c^ Estimates are average total months.

**TABLE 3:** Proportions and frequencies of second birth maternal risk factors and gestational age categories stratified by maternal race and medically indicated first birth.

Birth characteristics	First birth clinically indicated						First birth not clinically indicated					
	All mothers		White mothers		Black mothers		All mothers		White mothers		Black mothers	
Prev C‐section	24,497	(39.9%)	15,837	(38.9%)	8660	(41.6%)	3273	(11.5%)	1951	(11.2%)	1322	(12.0%)
Mother smoked tobacco	3377	(5.5%)	2776	(6.8%)	601	(2.9%)	1355	(4.8%)	1058	(6.1%)	297	(2.7%)
Mother used alcohol, drugs or other during pregnancy	635	(1.0%)	369	(0.9%)	266	(1.3%)	354	(1.3%)	149	(0.9%)	205	(1.9%)
Urinary tract infection	292	(0.5%)	151	(0.4%)	141	(0.7%)	157	(0.6%)	46	(0.3%)	111	(1.0%)
*Non‐UTI infection*	3157	(5.1%)	1419	(3.5%)	1738	(8.4%)	1679	(5.9%)	657	(3.8%)	1022	(9.3%)
Iron deficiency anaemia	9913	(16.1%)	4767	(11.7%)	5146	(24.7%)	4300	(15.1%)	1727	(9.9%)	2573	(23.5%)
Diabetes, any	3741	(6.1%)	2549	(6.3%)	1192	(5.7%)	1445	(5.1%)	924	(5.3%)	521	(4.8%)
Hypertension, any	7709	(12.5%)	4646	(11.4%)	3063	(14.7%)	2797	(9.8%)	1509	(8.7%)	1288	(11.7%)
Pregnancy complicating mental disorder	505	(0.8%)	405	(1.0%)	100	(0.5%)	216	(0.8%)	146	(0.8%)	70	(0.6%)
Preterm birth	5877	(9.6%)	3134	(7.7%)	2743	(13.2%)	3697	(13.0%)	1734	(9.9%)	1963	(17.9%)
Early preterm birth (< 32w)	762	(1.2%)	344	(0.8%)	418	(2.0%)	562	(2.0%)	199	(1.1%)	363	(3.3%)
Late preterm birth (32–36w)	5115	(8.3%)	2790	(6.9%)	2325	(11.2%)	3135	(11.0%)	1535	(8.8%)	1600	(14.6%)
Early term birth (37–38w)	16,279	(26.5%)	10,129	(24.9%)	6150	(29.6%)	8100	(28.5%)	4785	(27.4%)	3315	(30.2%)
Total births	61,458	(100%)	40,661	(100%)	20,797	(100%)	28,423	(100%)	17,452	(100%)	10,971	(100%)

*Note:* The analytical sample is limited to births from mothers whose first two consecutive births were live and observable in Georgia between 2011 and 2020. All measures except for previous caesarean delivery are exclusively derived from delivery hospital discharge records. Proportions in parentheses calculated for total women in the column group and do not sum to 100%. Information from both birth and hospital discharge records was used to distinguish delivery subtype as indicated, or not. Indicated births were those with no premature rupture of membranes (PROM) or premature labor or tocolysis, but with documentation of artificial rupture of membranes, medical or surgical induction, or caesarean delivery and no onset of labor (Appendices S2 and S3).

Adjusted models (Table 4) show patterns in gestational age categories consistent with the unadjusted estimates (Table 1). NH Black mothers who experienced a *first* preterm birth < 32 weeks had an RRR of 19.08 (95% CI 14.48, 24.98) for a second birth at < 32 weeks, relative to the reference group, NH White mothers who had *first* full‐term births. The comparable estimate for a NH White mother with a *first* preterm birth at < 32 weeks was substantially smaller at 10.17 (95% CI 7.00, 14.78). Similarly, RRRs for *second* births at 32–36 weeks after a *first* preterm birth < 32 weeks were higher for NH Black (RRR: 5.44 (95% CI 4.51, 6.57)) than NH White mothers (RRR: 3.82 (95% CI 3.00, 4.86)).

**TABLE 4:** Relative risk ratios for second birth gestational age category conditional on maternal race and first birth gestational age category.

Gestational week at delivery of 1st birth, by maternal race	Gestational week at delivery of the 2nd birth							
	Before 32		32–36		37–38		39–44	
White mother								
Before 32	10.17	(7.00, 14.78)	3.82	(3.00, 4.86)	1.86	(1.53, 2.27)	1.00	Reference
32–36	2.88	(2.20, 3.78)	3.71	(3.36, 4.10)	2.06	(1.92, 2.22)	1.00	Reference
37–38	1.73	(1.41, 2.11)	2.07	(1.92, 2.22)	1.81	(1.73, 1.90)	1.00	Reference
Black mother								
Before 32	19.08	(14.58, 24.98)	5.44	(4.51, 6.57)	2.18	(1.85, 2.58)	1.00	Reference
32–36	6.79	(5.43, 8.50)	4.32	(3.87, 4.82)	2.13	(1.95, 2.32)	1.00	Reference
37–38	3.53	(2.90, 4.30)	2.88	(2.64, 3.13)	1.92	(1.81, 2.04)	1.00	Reference
39–44	2.03	(1.70, 2.43)	1.65	(1.53, 1.78)	1.31	(1.25, 1.38)	1.00	Reference
Sample size								89,881

*Note:* Relative risk ratios calculated using multinomial logit models; referent outcome second full term birth. Excluded reference category for combinations of maternal race and first birth gestational age categories is NH White mothers with first full term births. The analytical sample is limited to births from mothers whose first two consecutive births were live and observable in Georgia between 2011 and 2020. Model includes covariates for: maternal age (ref: 35plus), educational attainment (ref: some college), birthplace (ref: USA), same paternal characteristics between 1st and 2nd birth separately for race, ethnicity and year of birth, Medicaid coverage, interpregnancy interval, previous c‐section, smoking/substance use status, maternal hypertension, diabetes and anaemia, maternal infection (UTI and non‐UTI), maternal mental health and year fixed effects.

Smaller but notable disparities were also seen for mothers with a *first* preterm birth at 32–36 weeks. Specifically, the RRRs for a second preterm birth < 32 or 32–36 weeks for NH Black mothers were 6.79 (95% CI 5.43, 8.50) and 4.32 (95% CI 3.87, 4.82), respectively, but for NH White mothers were 2.88 (95% CI 2.20, 3.78) and 3.71 (95% CI 3.36, 4.10), respectively. Relative to NH White mothers with a *first* full‐term birth, the likelihood of a *second* early term birth after a *first* preterm birth was smaller and similar in magnitude for mothers of both races with RRRs around 2.

Importantly, NH Black mothers who experienced a first early term birth were also more likely to experience *second* preterm births. Specifically, the RRRs for NH Black and White mothers experiencing a preterm birth at < 32 weeks after a *first* early term birth were respectively 3.53 (95% CI 2.90, 4.30) and 1.73 (95% CI 1.41, 2.11) whereas the RRRs for mothers experiencing a preterm birth at 32–36 weeks after a *first* early term birth were respectively 2.88 (95% CI 2.64, 3.13) and 2.07 (95% CI 1.92, 2.22). Differences in the likelihood of recurrent early term birth across NH Black and White mothers were smaller and more similar in magnitude (RRR: 1.92 (95% CI 1.81, 2.04) and 1.81 (95% CI 1.73, 1.90) respectively). Even NH Black mothers who experience a full term *first* birth were ultimately 2.03 times more likely (95% CI 1.7, 2.43) to experience a subsequent preterm birth at < 32 weeks. *Second* births at 32–36 weeks or early term were also more likely among NH Black mothers with full term *first* births than NH White mothers.

In sensitivity analyses excluding mothers with clinically indicated *first* births (Table 5), RRRs generally increased slightly, particularly for NH Black mothers. For example, RRRs for recurrent preterm birth at < 32 weeks for NH Black mothers increased from 19.08 (Table 4) to 20.12 (95% CI 13.82, 29.28) but were largely unchanged for NH White mothers from 10.17 to 10.08 (95% CI 6.03, 16.84), respectively. Overall, the RRR estimates for the smaller sample of births to women with non‐indicated *first* births reflect similar patterns in gestational age outcomes across NH White and NH Black mothers as the RRR estimates from the full sample (Table 4).

**TABLE 5:** Relative risk ratios for second birth gestational age category conditional on maternal race and first birth gestational age category, excluding mothers with indicated first births.

Gestational week at delivery of 1st birth, by maternal race	Gestational week at delivery of the 2nd birth							
	Before 32		32–36		37–38		39–44	
White mother								
Before 32	10.08	(6.03, 16.84)	4.34	(3.17, 5.95)	2.24	(1.74, 2.89)	1.00	Reference
32–36	3.57	(2.49, 5.14)	4.24	(3.67, 4.90)	2.31	(2.08, 2.56)	1.00	Reference
37–38	1.61	(1.11, 2.33)	2.29	(2.01, 2.62)	1.81	(1.67, 1.97)	1.00	Reference
Black mother								
Before 32	20.12	(13.82, 29.28)	5.35	(4.21, 6.79)	2.19	(1.78, 2.68)	1.00	Reference
32–36	8.02	(5.71, 11.26)	4.91	(4.17, 5.78)	2.34	(2.06, 2.65)	1.00	Reference
37–38	3.61	(2.56, 5.09)	3.16	(2.73, 3.67)	1.99	(1.79, 2.20)	1.00	Reference
39–44	2.29	(1.66, 3.15)	1.80	(1.57, 2.06)	1.20	(1.10, 1.32)	1.00	Reference
Sample size								28,423

*Note:* Relative risk ratios calculated using multinomial logit models; referent outcome second full term birth. Excluded reference category for combinations of maternal race and first birth gestational age categories is NH White mothers with first full term births. The analytical sample is limited to births from mothers whose first two consecutive births were live and observable in Georgia between 2011 and 2020, additionally excluding mothers with indicated first births. Indicated births were those with no premature rupture of membranes (PROM) or premature labor or tocolysis, but with documentation of artificial rupture of membranes, medical or surgical induction, or caesarean delivery and no onset of labor (Appendices S2 and S3). Model includes covariates for: maternal age (ref: 35plus), educational attainment (ref: some college), birthplace (ref: USA), same paternal characteristics between 1st and 2nd birth separately for race, ethnicity and year of birth, Medicaid coverage, interpregnancy interval, previous c‐section, smoking/substance use status, maternal hypertension, diabetes and anaemia, maternal infection (UTI and non‐UTI), maternal mental health and year fixed effects.

## 4 Comment

### 4.1 Principal Findings

Using data from Georgia, we confirm that mothers who experience *first* preterm births are at increased risk of subsequent preterm births, that there is a gradient for increased risk for recurrent preterm birth among mothers with the earliest preterm births, and that in all cases this risk is more likely for NH Black women compared to NH White women. We also confirm findings in a small but growing literature that shows elevated risk for subsequent preterm birth also applies to mothers with first early term births [12, 13, 14, 15, 18], an often overlooked group, and extend this prior literature with new findings that show that racial disparities in risk of subsequent preterm birth persist in this group as well. Lastly, we found that NH Black mothers with previous full term births were 1.3 to 2.03 times more likely to experience subsequent shortened gestational births relative to NH White mothers with full term births.

### 4.2 Strengths of the Study

In this study, we leveraged recent linked hospital and birth record data to perform a population‐wide analysis of mothers' likelihood of experiencing *second* preterm births. The maternal identifiers in the data allowed us to stratify mothers by maternal race and past live birth outcomes while the hospital discharge records allowed us to observe birth‐specific characteristics. Collectively, we were able to measure the association between gestational age across births while also accounting for confounding factors that allowed us to produce more nuanced insights into the underlying associations. Analysis of birth outcomes using mothers' gestational age trajectories over time were also especially useful for evaluating race disparities. There is a broad array of complex causal factors that conceptually explain racial disparities in birth outcomes [21, 32, 33, 34, 35, 36]. Studying racial disparities using longitudinal data offers a dynamic perspective to the effects of living in a racialised society on minority health [37].

Methodologically, we also used multinomial logit models to jointly estimate mothers' subsequent gestational age categories unlike most of the existing literature [13, 19, 20, 31]. The models ensure probabilities of gestational age categories correctly sum to 1 and allowed us to compare estimates by maternal race using a common benchmark. Separate race‐specific models do not allow such comparisons [29].

Studying Georgia offers important insights and is also a strength of this study given its historical and geographic context. Relative to other states, Georgia has poor maternal and infant health outcomes that reflect health inequities [24]. Georgia also has had a significant and evolving role in US history. Before the Civil War ended slavery in the US, Georgia had a substantial slave‐based economy and actively resisted post‐war reforms; however, over time Georgia also became a center for the Civil Rights Movement [38]. Although these historical events may appear irrelevant to contemporary population health, there is empirical evidence linking place‐based historical legacy of slavery to contemporary spatial health inequities [36, 39]. The racial disparities in gestational age outcomes we observed likely reflect present‐day inequities and historical antecedents. Policy reforms are essential to address persistent racial disparities in birth outcomes. Georgia has not expanded Medicaid eligibility for childless low‐income adults, a policy change that could substantially improve access to care and reduce interpregnancy risks for vulnerable mothers. Extending Medicaid coverage could mitigate risk for preterm and early term births through improved management of chronic conditions and enhanced interpregnancy care [24, 40, 41, 42, 43, 44].

### 4.3 Limitations

Our use of data from a single‐state may not generalise to other states with different racial compositions, healthcare systems, or policy environments [13, 45]. Second, our use of administrative record data has limitations. Maternal administrative records often reflect information on mothers' health at a point in time which may not reflect the totality of maternal health during pregnancy. Use of diagnostic codes to identify maternal risk factors at delivery may exclude other health factors not diagnosed by attending medical providers during delivery. Third, we excluded mothers with key missing data (e.g., race or ethnicity), which may have introduced selection bias. Fourth, our data only capture pregnancies that resulted in a live birth and do not reflect spontaneous abortions, stillbirths, or terminated pregnancies, potentially underestimating racial disparities in reproductive health more broadly. Finally, we acknowledge the previous research supporting shared etiologies among clinically indicated and spontaneous births and the publication of new preterm birth taxonomy systems that better reflect the causal mechanisms underlying preterm births [17, 46, 47]; however, our data did not allow for application of those systems. Despite these limitations, the large population‐level dataset allowed us sufficient data to contribute to the limited literature focused on population‐wide longitudinal analysis of birth outcomes to mothers from minority subpopulations.

### 4.4 Interpretation

This study contributes to the separate literatures on recurrent preterm births, early term births and race disparities in preterm births as well as the smaller subset of studies at the intersection of these literatures [2, 3, 4, 5, 6, 7, 8, 9, 13, 14, 15, 16, 18, 19, 20, 21]. Our findings that past short gestational age (preterm or early term) is still linked to subsequent early births in recent data demonstrates that in spite of the invaluable aforementioned research to understand factors that make some women at greater risk for preterm births, unobserved factors reflected in maternal birth history still matter for identifying expectant mothers at risk for early births. Our new evidence that NH Black mothers are more likely to experience *second* preterm births after *first* early term births suggests that including history of early term births along with preterm births to risk screenings for expectant mothers may offer public health benefits that are especially impactful for NH Black mothers.

Conceptually, our evidence that NH Black women experienced elevated risk for recurrent early births suggests that some of the unobserved mechanisms that cause mothers to experience early births are sensitive to race, a social construct. In our supplementary analysis where we focus on the intersection of racial disparities in shortened gestational age and preterm phenotype, our findings support hypotheses that link structural inequities with biological vulnerability. Evidence of persistent disparities in both clinically indicated and non‐indicated births highlight the multifactorial pathways by which race can affect birth outcomes [21]. With respect to spontaneous births, our findings of race disparities in subsequent births among mothers with non‐indicated *first* births highlight the roles of racism, stress, inflammation and neuroendocrine dysfunction [21, 32, 34, 48]. Furthermore, the higher likelihoods of preterm *second* births experienced by NH Black mothers after both early term and full term births we observed in our data are consistent with theoretical models, such as weathering, which posits repeated exposure to social and structural adversity contributes to declining reproductive health outcomes over time [49]. NH Black mothers' relatively accelerated decline in birth outcomes in our data may represent the cumulative effects of these exposures.

## 5 Conclusions

Our findings reinforce that past preterm birth—whether spontaneous or clinically indicated—remains a strong predictor of subsequent preterm birth, especially among NH Black mothers. However, the risk of shortened gestation also extends to NH Black mothers with first early‐term or even full‐term births and is not fully explained by sociodemographic or clinical factors. These findings suggest the importance of incorporating longitudinal birth history—and racial context—into preterm and early‐term birth risk assessments [18]. Persistent disparities in gestational age at delivery underscore the need for comprehensive, equity‐focused maternal health interventions across the reproductive life course.

## Author Contributions

A.L.D. and P.K.C. developed the study objective and design. P.K.C., M.R.K. and M.D. prepared the data for analysis. P.K.C. and M.D. analyzed data and interpreted outcomes. P.K.C. wrote the first draft. M.R.K., E.K.A., A.L.D. and M.D. reviewed and revised the manuscript. E.K.A. was a major contributor in writing the manuscript. All authors read and approved the final manuscript.

## Conflicts of Interest

The authors declare no conflicts of interest.

## Supporting information


**Data S1:** ppe70083‐sup‐0001‐DataS1.zip.

## Data Availability

The data that support the findings of this study are available from the Georgia Department of Public Health. Restrictions apply to the availability of these data, which were used under licence for this study. Requests for use of these restricted‐access data can be made at https://dph.georgia.gov/phip‐data‐request.
