# Supplementary material for: Disparities in Patterns of Preterm and Early Term Second Births Among Non‐Hispanic Black and White Mothers
Source: Paediatr Perinat Epidemiol. 2025 Nov 16;40(1):19–30. doi: 10.1111/ppe.70083 (PMC12853229; doi:10.1111/ppe.70083)
Supplement: Supplementary file 1 — Data S1: ppe70083‐sup‐0001‐DataS1.zip. [file PPE-40-19-s001.zip › ppe70083-sup-0002-Supinfo02.docx]

**Appendix 1: Sample Flow Diagram**

**
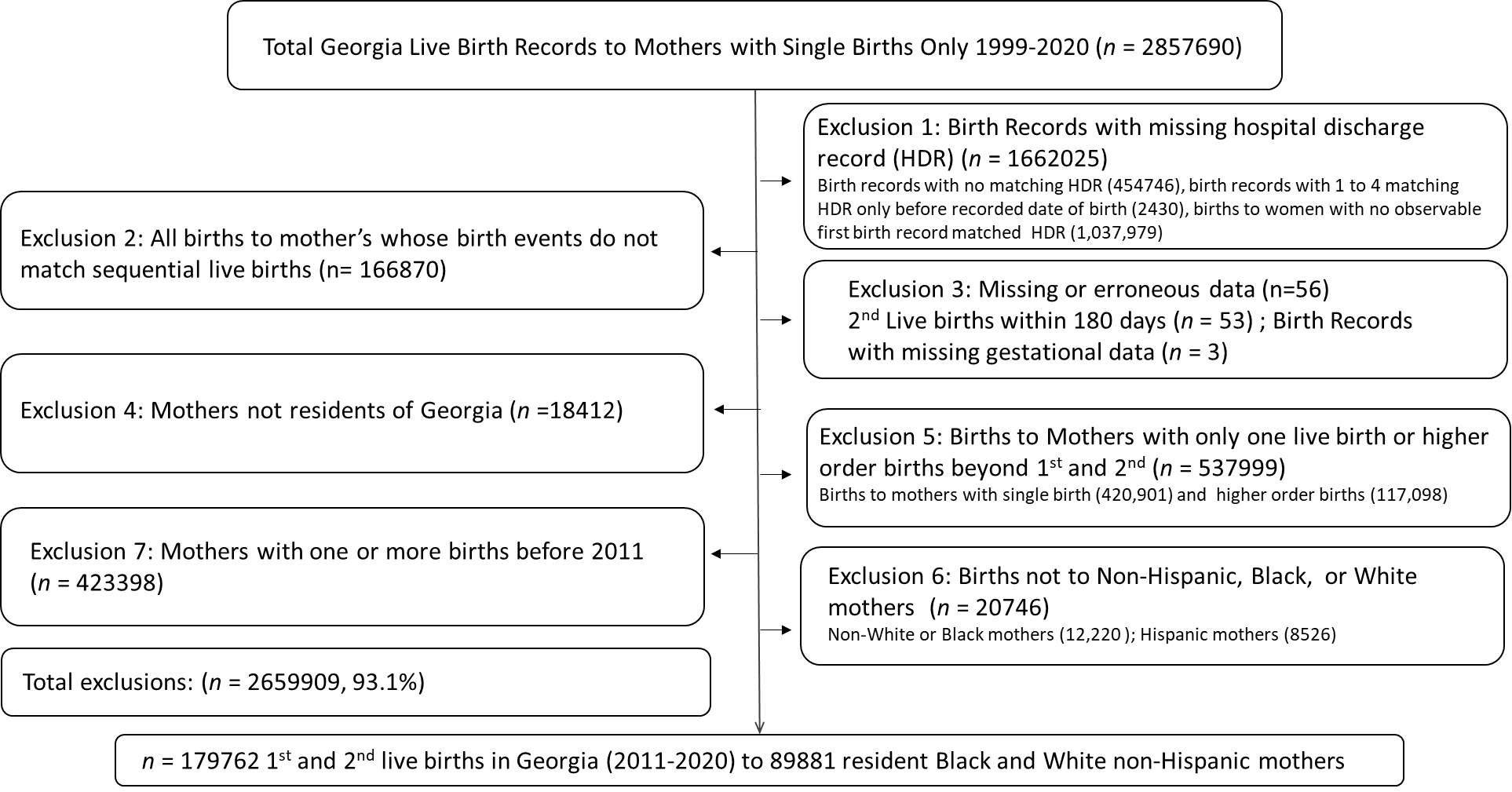
**

**Appendix 2: Variable Construction**

**mom_black, mom_white**

Birth record race variables

Value Label

-1 Unknown

1 White

2 Black or African American

3 Asian

4 American Indian/Alaska Native

5 Native Hawaiian / Pacific Islander

6 Multiracial

**interval_imp, short_imputed,** replace missing interval with hand calculated using dob. Short implies < 18 months since last birth

by id: generate interval_byhand= dob -L.dob

replace interval_byhand = interval_byhand/30

generate interval_imp = interval_N

replace interval_imp = interval_byhand if interval_N==-1 & i>1

generate short_imputed = interval_imp<18 & i>1

**Medicaid, Medicaid_2,** - variable generated from HDR for any payer

Medicaid =1 if the primary, secondary or third payer variables from the HDR data (below)

was 1- Medicaid managed care, 2 Medicaid, 5 Medicaid Applicants, 6 Georgia Better Health, 18 Peachcare or 19 State Health Benefit Plan

*Exclude 10 Medicare, 11 other Government Assistance, 14 Medicare Managed care

-1 Unknown

1 Medicaid Managed Care

2 Blue Cross / Blue Shield

3 Champus

4 Medicaid

5 Medicaid Applicants

6 Georgia Better Health

7 HMO/Managed Care

8 Commercial Insurance

9 Other non-specified Managed Care

10 Medicare

11 Other Government Assistance

12 Other

13 Self Pay

14 Medicare Managed Care

15 Workers Compensation

16 PPO

17 POS

18 PeachCare

19 State Health Benefit Plan

**tobacco_any and tobacco_HD,** smoker if either HDR or BC says so

Created variable **smoker**, gen smoker = tobacco_any==1 | tobacco_HD==1

**spontaneous indicated,** for delivery type derived from a mix of variables that can be ignored otherwise

Spontaneous if

- Premature rupture of membranes (prom_HD==1) or (PROM==1), or
- Premature labor: any of eod_37_HD, eol_37_HD, or eod_37_39_HD ==1, or
- Tocolysis administered: tocolysis==1

gen spontaneous = 0

replace spontaneous = 1 if prom_HD==1 | PROM==1 // PROM

replace spontaneous = 1 if eod_37_HD==1 | eol_37_HD==1 | eod_37_39==1 // premature labor

replace spontaneous = 1 if tocolysis==1 // tocolytic medication

replace spontaneous = 1 if (spontaneous==0 & indicated==0) & (all_ind_HD==0 & stimulation==0 & induction==0 & dm_c_HD==0)

Indicated PROM==0 AND,

- Medical induction, surgical induction or artificial rupture of membranes: all_ind_HD, stimulation, or induction ==1, or
- Current C-section: dm_c_HD (Derived from HDR), CS (from birth records)

gen indicated = 0

replace indicated = 1 if spontaneous == 0 and (all_ind_HD==1 | stimulation==1 | induction==1) // not spontaneous AND surgical induction or artificial membrane rupture

replace indicated = 1 if dm_c_HD==1 // c-section

replace indicated = 0 if spontaneous == 1 // can’t be indicated if spontaneous

**Appendix 3: ICD**

**ICD Code sources:**

https://www.icd10data.com/ICD10CM/Codes

Differentiating Between Maternal Pre-Gestational Diabetes and Gestational Diabetes <http://mchp-appserv.cpe.umanitoba.ca/viewConcept.php?printer=Y&conceptID=1451>

“What are the documentation requirements for vaginal deliveries?” <https://www.acog.org/practice-management/coding/coding-library/documentation-requirements-for-vaginal-deliveries>

“ICD-10-PCS Coding Advice for Labor Inductions” <https://www.cmqcc.org/sites/default/files/ICD10%20Labor%20Induction%20FINAL_8.3.16.pdf>

New DRG codes for C-sections and vaginal deliveries <https://www.mhswi.com/newsroom/New-DRG-codes-for-C-sections-and-vaginal-deliveries.html>

Obstetrics Coding and Documentation Reference Guide

<https://providers.bcbsal.org/portal/documents/10226/306297/Obstetrics+Coding+and+Documentation+Reference+Guide/8f5f1b65-1fd2-49a5-8708-6819a162098e>

See supplemental file for codes used in Yang, J., Baer, R. J., Berghella, V., Chambers, C., Chung, P., Coker, T., Currier, R. J., Druzin, M. L., Kuppermann, M., & Muglia, L. J. (2016). Recurrence of preterm birth and early term birth. Obstetrics and gynecology, 128(2), 364.

Lydon-Rochelle, M. T., Holt, V. L., Cárdenas, V., Nelson, J. C., Easterling, T. R., Gardella, C., & Callaghan, W. M. (2005). The reporting of pre-existing maternal medical conditions and complications of pregnancy on birth certificates and in hospital discharge data. American journal of obstetrics and gynecology, 193(1), 125-134.

Lydon‐Rochelle, M. T., Holt, V. L., Nelson, J. C., Cárdenas, V., Gardella, C., Easterling, T. R., & Callaghan, W. M. (2005). Accuracy of reporting maternal in‐hospital diagnoses and intrapartum procedures in Washington State linked birth records. Paediatric and Perinatal Epidemiology, 19(6), 460-471.

**Appendix 4 Missing Covariates and Multiple Imputation**

| Characteristic |  | Missing Obs | |
| --- | --- | --- | --- |
| Gestation Week of 2nd Birth |  | 0 | (0.0%) |
| Previous Gestation |  | 0 | (0.0%) |
| Maternal Race |  | 0 | (0.0%) |
| Maternal Age |  |  |  |
| *less than 18 years of age* |  | 0 | (0.0%) |
| between 18-34 years of age |  | 0 | (0.0%) |
| 35 years or older |  | 0 | (0.0%) |
| Mother not born in the US |  | 623 | (0.7%) |
| Maternal Education |  |  |  |
| less than high school diploma |  | 580 | (0.7%) |
| has high school diploma |  | 580 | (0.7%) |
| has some college education or more |  | 580 | (0.7%) |
| Faternal Race |  | 24,097 | (26.8%) |
| Faternal Birth Year |  | 18,394 | (20.5%) |
| Faternal Ethnicity |  | 7,136 | (7.9%) |
| Mother Maritial Status |  | 59 | (0.1%) |
| Mother Medicaid |  | 0 | (0.0%) |
| Birth Interval Months |  | 0 | (0.0%) |
| Previous C-section |  | 0 | (0.0%) |
| Mother Smoked Tobacco |  | 0 | (0.0%) |
|  |  |  |  |
| Mother used Alcohol, Drugs, or Other during Pregnancy |  | 0 | (0.0%) |
| Urinary Tract Infection |  | 0 | (0.0%) |
| Non-UTI Infection |  | 0 | (0.0%) |
| Iron Deficiency Anemia |  | 0 | (0.0%) |
| Diabetes, any |  | 0 | (0.0%) |
| Hypertension, any |  | 0 | (0.0%) |
| Pregnancy Complicating Mental Disorder |  | 0 | (0.0%) |
| Total Mothers |  | 89,881 | (100%) |

Multivariate imputation Imputations = 10

Chained equations added = 10

Imputed: m=1 through m=10 updated = 0

Initialization: monotone Iterations = 100

burn-in = 10

mom_born_not~A: logistic regression

dad_race_same: logistic regression

dad_birthyea~e: logistic regression

dad_ethnic_s~e: logistic regression

married: logistic regression

mom_ed_3cat: multinomial logistic regression

------------------------------------------------------------------

| Observations per m

|----------------------------------------------

Variable | Complete Incomplete Imputed | Total

-------------------+-----------------------------------+----------

mom_born_not~A | 89258 623 623 | 89881

dad_race_same | 65784 24097 24097 | 89881

dad_birthyea~e | 71487 18394 18394 | 89881

dad_ethnic_s~e | 82745 7136 7136 | 89881

married | 89822 59 59 | 89881

mom_ed_3cat | 89301 580 580 | 89881

------------------------------------------------------------------

(Complete + Incomplete = Total; Imputed is the minimum across m

of the number of filled-in observations.)
