# Supplementary material for: Disparities in Patterns of Preterm and Early Term Second Births Among Non‐Hispanic Black and White Mothers
Source: Paediatr Perinat Epidemiol. 2025 Nov 16;40(1):19–30. doi: 10.1111/ppe.70083 (PMC12853229; doi:10.1111/ppe.70083)
Supplement: Supplementary file 1 — Data S1: ppe70083‐sup‐0001‐DataS1.zip. [file PPE-40-19-s001.zip › ppe70083-sup-0001-Supinfo01.pptx]

## Slide 1
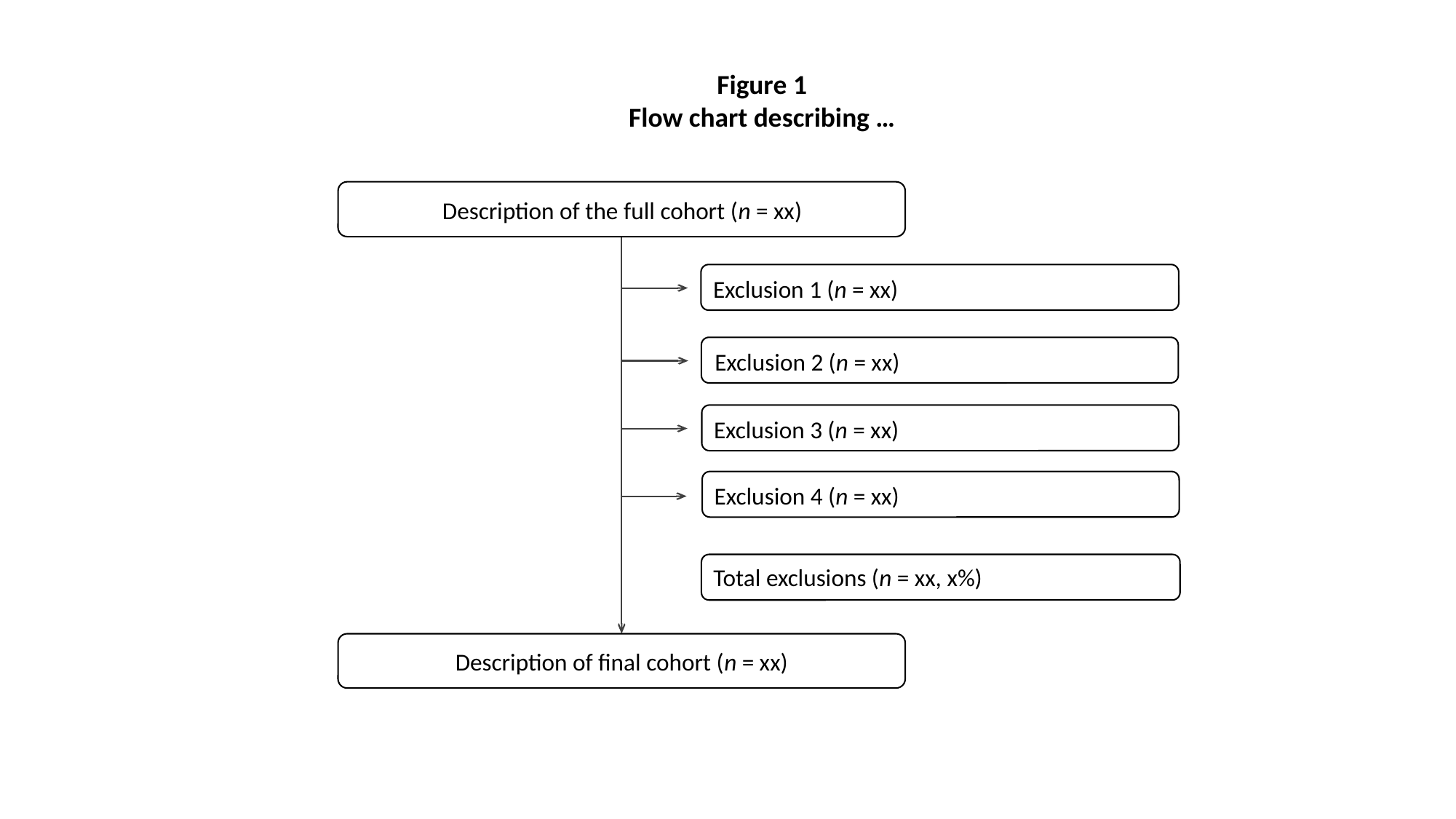

Figure 1
Flow chart describing …
Description of the full cohort (n = xx)
Exclusion 1 (n = xx)
Exclusion 2 (n = xx)
Exclusion 3 (n = xx)
Exclusion 4 (n = xx)
Total exclusions (n = xx, x%)
Description of final cohort (n = xx)
